# Repeated Continuous Theta-Burst Stimulation Modulates Resting-State EEG Microstate Dynamics in Athletes with Chronic Insomnia

**DOI:** 10.3390/brainsci16080841

**Published:** 2026-08-07

**Authors:** Xiao He, Bohan Cheng, Hui He, Li Zhao, Yan Li

**Affiliations:** 1 School of Sport Science, Beijing Sport University, Beijing 100084, China; 2023011858@bsu.edu.cn (X.H.); 2023011857@bsu.edu.cn (B.C.); zhaolispring@bsu.edu.cn (L.Z.); 2 Beijing Key Laboratory of Sports Performance and Skill Assessment, Beijing Sport University, Beijing 100084, China; 3 China Institute of Sport and Health Science, Beijing Sport University, Beijing 100084, China; hehui@bsu.edu.cn

**Keywords:** athletes, chronic insomnia, continuous theta-burst stimulation, EEG microstates, sleep quality, athletic performance

## Abstract

**Highlights:**

**What are the main findings?**

- A 7-day course of right dorsolateral prefrontal cTBS improved subjective sleep quality and sport-related performance in male athletes with chronic insomnia.
- Active cTBS selectively reorganized resting-state EEG microstate dynamics, increasing microstate C duration and contribution, reducing microstate A occurrence, and modulating directional transitions among microstates B, C, and D.

**What are the implications of the main findings?**

- cTBS may represent a brief, feasible, and non-pharmacological neuromodulatory approach for managing chronic insomnia in athletic populations.
- EEG microstate temporal properties and transition dynamics may provide sensitive electrophysiological markers for characterizing treatment-related brain-state reorganization associated with improvements in sleep and athletic performance.

**Abstract:**

Background: Chronic insomnia can impair recovery and performance in athletes. Continuous theta-burst stimulation (cTBS) is a brief neuromodulatory intervention, but its effects in athletes with chronic insomnia remain unclear. This study evaluated whether right dorsolateral prefrontal cTBS improves sleep quality and athletic performance and alters resting-state EEG microstate dynamics. Methods: In this randomized, sham-controlled trial, 35 male athletes with chronic insomnia received active cTBS or sham stimulation once daily for 7 days; usable EEG data were obtained from 32 participants. Sleep quality was assessed using the Pittsburgh Sleep Quality Index (PSQI). Countermovement jump, 50 m sprint, and Illinois Agility Test performance were assessed before and after intervention. Resting-state electroencephalography (EEG) microstate temporal parameters and directional transition probabilities were analyzed. Results: Active cTBS produced a greater reduction in PSQI global score than sham stimulation. Active stimulation increased microstate C duration and contribution and reduced microstate A occurrence. Directional transitions among microstates B, C, and D were also altered, with largely concordant findings in ΔTM sensitivity analyses. Active cTBS improved countermovement jump height, 50 m sprint performance, and agility, whereas sham stimulation improved sprint performance only. Conclusions: A 7-day course of right dorsolateral prefrontal cTBS improved subjective sleep quality and sport-related performance in athletes with chronic insomnia. These improvements were accompanied by selective changes in resting-state EEG microstate temporal parameters and transition probabilities. Given the exploratory nature of the EEG findings, these microstate changes should be interpreted as potential electrophysiological correlates of treatment response rather than evidence of a causal mechanism.

## 1. Introduction

Sleep disturbance has emerged as a major public health concern, with sleep-related complaints affecting a substantial proportion of the adult population worldwide. Athletes may be particularly susceptible because they are exposed not only to the psychosocial stressors encountered by the general population, but also to sport-specific demands, including intensive training schedules, competition-related pressure, travel, and insufficient recovery [1,2,3]. Repeated exposure to high training loads may disrupt the balance between neural activation and recovery, alter neuroendocrine regulation, and promote a state of persistent physiological and cognitive hyperarousal, thereby impairing both sleep initiation and sleep maintenance. Consistent with this view, epidemiological studies have reported a higher prevalence of sleep disturbances in athletes than in age-matched non-athletic populations, with the overall risk estimated to be approximately 1.5-fold greater and 22–26% of affected athletes experiencing severe symptoms. Importantly, inadequate sleep may compromise physiological recovery, training adaptation, cognitive function, and sport-specific performance, while also increasing vulnerability to fatigue and injury [1,3,4]. Therefore, the development of effective, feasible, and athlete-oriented interventions for chronic sleep disturbance is of considerable importance to both sports neuroscience and clinical practice.

Pharmacotherapy remains a common treatment option for insomnia, with benzodiazepines and related sedative–hypnotic agents frequently used to reduce sleep-onset latency and nocturnal awakenings. However, prolonged or inappropriate use may be accompanied by residual daytime sedation, cognitive impairment, tolerance, dependence, and alterations in normal sleep architecture [5]. These limitations are particularly relevant to athletes, for whom psychomotor vigilance, reaction time, motor coordination, and recovery quality are essential to both training and competition. Medication use in competitive sport may also require careful consideration of anti-doping regulations, potential interactions with other treatments, and therapeutic use documentation [6]. Consequently, increasing attention has been directed toward non-pharmacological interventions that can improve sleep while minimizing systemic adverse effects.

Among non-invasive neuromodulation approaches, repetitive transcranial magnetic stimulation (rTMS) has demonstrated therapeutic potential for insomnia by modulating cortical excitability and sleep-related neural networks [7]. Nevertheless, conventional rTMS protocols typically require relatively long stimulation sessions and repeated treatment visits, which may limit their practicality in athletic populations with demanding training schedules, frequent competition, and restricted recovery periods. These practical constraints highlight the need for shorter and more efficient neuromodulation protocols that can be integrated into sports training and recovery programs [7].

Among patterned rTMS protocols, continuous theta-burst stimulation (cTBS) has attracted increasing attention because of its high time efficiency and capacity to induce lasting neuroplastic effects [7,8]. A standard cTBS train consists of bursts of three pulses delivered at 50 Hz and repeated at a theta frequency of 5 Hz. When applied over the motor cortex, cTBS commonly produces a transient reduction in cortical excitability, an effect that has been associated with long-term depression-like synaptic plasticity. However, its physiological effects may vary according to the stimulation target, dose, intensity, and individual neurobiological characteristics [9]. By modulating cortical excitability and synaptic plasticity, cTBS may alter the functional organization and temporal coordination of distributed brain networks implicated in arousal regulation, cognitive control, and sleep.

Compared with conventional rTMS, cTBS can be delivered within a substantially shorter period and at a relatively low stimulation intensity, while its neuromodulatory effects may persist beyond the stimulation session. These features make cTBS particularly attractive for athletic populations, in whom prolonged treatment sessions may be difficult to accommodate alongside training, competition, and recovery schedules [7,8]. Although preliminary evidence suggests that cTBS may alleviate insomnia-related symptoms, its underlying neural mechanisms remain insufficiently understood, particularly in athletes, whose sleep disturbances may arise from a distinctive combination of training load, physiological stress, cognitive arousal, and recovery demands. Characterizing cTBS-induced alterations in large-scale brain network dynamics may therefore provide important mechanistic insight into its potential therapeutic effects on chronic insomnia in athletes [10,11].

Resting-state electroencephalography (EEG) provides a non-invasive approach for examining spontaneous neural activity with high temporal resolution and is therefore well suited to characterizing the rapid brain dynamics that may underlie insomnia and its response to neuromodulation. In particular, EEG microstate analysis segments continuous brain activity into a sequence of quasi-stable scalp topographies that persist for tens of milliseconds and are thought to reflect transient configurations of large-scale neural networks. Rather than focusing solely on regional oscillatory activity, microstate analysis captures the temporal organization of whole-brain neural states through parameters such as mean duration, occurrence, contribution, and transition probabilities [12]. These measures provide complementary information regarding the stability, predominance, and switching patterns of distributed brain states. Recent evidence has demonstrated that a single session of theta-burst stimulation can induce frequency-specific modulation of resting-state EEG microstates in healthy individuals [13], supporting the sensitivity of brain-state dynamics to non-invasive brain stimulation. However, whether repeated therapeutic cTBS reshapes EEG microstate dynamics following a 7-day repeated treatment course in athletes with chronic insomnia remains unknown. Accordingly, investigating cTBS-induced alterations in EEG microstate dynamics may help clarify how neuromodulation reshapes the temporal architecture of resting-state brain activity in athletes with chronic insomnia and whether these neural changes are associated with improvements in sleep quality and athletic performance [14].

Therefore, the present study aimed to determine whether a 7-day course of cTBS applied over the right dorsolateral prefrontal cortex could improve sleep quality and sport-related performance in athletes with chronic insomnia. Resting-state EEG microstate analysis was further employed to examine intervention-related changes in microstate duration, occurrence, contribution, and transition dynamics, thereby identifying potential neural signatures underlying the effects of cTBS.

## 2. Materials and Methods

### 2.1. Participant Recruitment and Assessment

This randomized, sham-controlled, participant- and assessor-blinded study recruited male athletes with chronic insomnia from Beijing Sport University. An a priori power analysis was conducted in G*Power 3.1 (Universität Düsseldorf, Düsseldorf, Germany) based on primary sleep-related outcomes from previous rTMS/cTBS studies in insomnia populations. With an estimated effect size of f = 0.27, a mixed-design ANOVA including group (active vs. sham) as the between-subject factor and time (pre- vs. post-intervention) as the within-subject factor, two-tailed α = 0.05, and power = 0.80, at least 15 participants per group were required. Eligible participants were 18–24 years old, right-handed, had a Pittsburgh Sleep Quality Index (PSQI) score > 7, and scored < 7 on both the Hamilton Depression Rating Scale and Hamilton Anxiety Rating Scale [15]. Chronic insomnia was diagnosed according to the International Classification of Sleep Disorders, Third Edition, using a structured clinical interview. Exclusion criteria included neurological or psychiatric disorders, use of sleep medication within the previous 2 weeks, and contraindications to transcranial magnetic stimulation [16,17,18].

Thirty-six athletes were recruited, and 35 were enrolled after screening and allocated to active cTBS or sham stimulation using a computer-generated randomization sequence. The study was approved by the Sports Science Experiment Ethics Committee of Beijing Sport University (No. 2025141H) and registered with the Chinese Clinical Trial Registry (ChiCTR2500109935). All participants provided written informed consent.

### 2.2. Sleep Quality Assessment: Pittsburgh Sleep Quality Index

Subjective sleep quality was assessed before and after the intervention using the PSQI, a validated 19-item self-report questionnaire yielding a global score of 0–21, with higher scores indicating poorer sleep quality. A score > 7 was considered indicative of clinically relevant sleep disturbance. The PSQI global score was designated as the primary sleep-related outcome [15].

### 2.3. cTBS Intervention

Continuous theta-burst stimulation was administered using a Yiruide magnetic stimulator (Yiruide Medical Equipment Co., Wuhan, China) over the right dorsolateral prefrontal cortex, corresponding to the F4 position of the international 10–20 EEG system. Stimulation intensity was set at 80% of the resting motor threshold. Participants received one session daily for 7 consecutive days, with each session consisting of three 600-pulse cTBS blocks, yielding a total of 1800 pulses [10,11]. For sham stimulation, the coil was tilted 90° relative to the scalp to preserve the acoustic and somatosensory characteristics of active stimulation while minimizing effective cortical stimulation. Participants and outcome assessors remained blinded to group allocation. No serious adverse events occurred during the intervention period [10].

### 2.4. EEG Data Acquisition

Five minutes of resting-state EEG were recorded between 19:00 and 22:00 using a 64-channel NeuSen W EEG system (Neuracle, Shanghai, China). Signals were sampled at 1000 Hz, with CPz used as the online reference and AFz as the ground electrode. A 0.1–40 Hz band-pass filter was applied during acquisition, and electrode impedances were maintained below 30 kΩ. Participants were instructed to remain seated, relaxed, awake, and motionless with their eyes closed throughout the recording.

### 2.5. EEG Preprocessing

EEG preprocessing was performed in MATLAB (R2021b, MathWorks, Natick, MA, USA) using EEGLAB (v2024.0, University of California, San Diego, CA, USA). Continuous recordings were band-pass filtered from 0.1 to 40 Hz, down-sampled to 250 Hz, and re-referenced to the common average [19]. Independent component analysis was conducted using the extended Infomax algorithm, and artifact-related components were identified based on ICLabel classification and visual inspection [20]. Components reflecting ocular, muscular, cardiac, or other non-neural activity were removed before subsequent microstate analysis. Three participants were excluded because of excessive EEG artifacts, resulting in a final EEG sample of 32 participants.

### 2.6. EEG Microstate Analysis

EEG microstate analysis was performed in MATLAB using EEGLAB and the EEGLAB Microstate Analysis Toolbox [21]. Global field power (GFP) was calculated across all scalp electrodes, and scalp maps at local GFP peaks were submitted to polarity-invariant modified k-means clustering. The number of microstate classes was predefined as four, corresponding to the canonical microstates A–D. These classes have been tentatively associated with auditory/language processing (A), visual processing (B), internally directed and salience-related processing (C), and attention and cognitive control (D), although these functional interpretations remain putative [22].

To ensure consistent labeling across participants, groups, and time points, individual microstate maps were matched to a common grand-mean template according to polarity-independent spatial correlations. The resulting template maps were then back-fitted to the continuous EEG recordings by assigning each sample to the microstate class showing the highest absolute spatial correlation [23].

For each microstate class, mean duration, occurrence, contribution, and mean GFP were extracted. Mean duration represented the average uninterrupted persistence of a microstate, occurrence represented the number of appearances per second, and contribution represented the proportion of the analyzed recording occupied by that microstate. Mean GFP reflected the average global field strength during periods assigned to each class. Global explained variance was calculated to quantify the proportion of topographic variance explained by the four-class solution, and the duration of retained artifact-free EEG was recorded as TotalTime [23].

Directional transition probabilities were derived from the observed transition matrix (OrgTM) to characterize switching among microstates A–D [21]. Transition direction was preserved; therefore, C to D and D to C were treated as distinct measures. Self-transitions were excluded because consecutive samples assigned to the same class were considered part of a single microstate segment [24]. As a sensitivity analysis, transition analyses were repeated using ΔTM values exported by the toolbox, representing the deviation of observed transition probabilities from their expected values [21,24]. Robustness was evaluated according to the consistency of the direction and magnitude of the Group × Time effects between the OrgTM and ΔTM analyses. All microstate parameters were averaged at the participant and condition levels for subsequent statistical analyses.

### 2.7. Sports Performance Assessment

Sports performance was assessed before and after the intervention using three standardized tests: countermovement jump (CMJ) height for lower-limb explosive power, 50 m sprint time for speed, and the Illinois Agility Test (IAT) for agility. All assessments were conducted at the same time of day under standardized conditions to minimize potential circadian effects [25,26].

### 2.8. Statistical Analysis

Statistical analyses were performed using SPSS Statistics (version 29.0, IBM, New York, NY, USA). For each outcome, a 2 × 2 mixed-design repeated-measures ANOVA was conducted, with Group (active cTBS vs. sham stimulation) as the between-subjects factor and Time (pre- vs. post-intervention) as the within-subject factor. No covariates were included in these models. Approximate normality of model residuals was assessed using Q–Q plots and the Shapiro–Wilk test, and homogeneity of variance was assessed using Levene’s test. Because the within-subject factor Time had only two levels, the sphericity assumption was inherently satisfied. For EEG outcomes, multiple testing was controlled using the Benjamini–Hochberg false discovery rate (FDR) procedure, with statistical significance defined as an FDR-adjusted *p* value < 0.05. FDR correction was applied separately to three analysis families of omnibus Group × Time interaction tests: (1) 16 class-specific microstate parameter tests comprising mean duration, occurrence, contribution, and mean GFP for microstates A–D; (2) 12 directional non-self transition-probability tests derived from the observed transition matrix (OrgTM); and (3) the corresponding 12 ΔTM transition tests included in the sensitivity analysis. The FDR procedure was applied to the omnibus Group × Time interaction *p* values within each analysis family. For the primary EEG analyses, Group × Time interactions that remained significant after FDR correction were followed by simple-effects comparisons based on estimated marginal means, with pairwise comparisons adjusted using the Bonferroni procedure. The ΔTM sensitivity analyses were conducted using the same mixed-design repeated-measures model and the same Benjamini–Hochberg FDR procedure as the primary OrgTM analyses. Significant FDR-adjusted Group × Time interactions in the ΔTM analyses were likewise followed by Bonferroni-adjusted simple-effects comparisons based on estimated marginal means. For non-EEG outcomes, significant Group × Time interactions were followed by the same simple-effects procedure. Effect sizes for omnibus effects were reported as partial eta squared (η^2^p). All statistical tests were two-sided, and statistical significance for analyses not subjected to FDR correction was set at *p* < 0.05.

## 3. Results

Of the 35 randomized participants (active cTBS, *n* = 18; sham, *n* = 17), all participants completed the 7-day intervention and the pre- and post-intervention sleep and sport-performance assessments. Three EEG datasets (active cTBS, *n* = 2; sham, *n* = 1) were excluded because of excessive artifacts, leaving 32 participants for the EEG analyses (active cTBS, *n* = 16; sham, *n* = 16). Participant flow is detailed in the CONSORT flow diagram [27] (Supplementary Material Figure S1).

### 3.1. Effects of cTBS on Subjective Sleep Quality

Repeated-measures ANOVA showed a significant Group × Time interaction for the PSQI global score (F(1, 30) = 16.45, *p* < 0.001, η^2^p = 0.354), indicating a greater improvement in subjective sleep quality following active cTBS than sham stimulation. No significant between-group difference was observed at baseline. Significant Group × Time interactions were also found for the PSQI components of subjective sleep quality, sleep latency, sleep duration, and sleep disturbances. In contrast, habitual sleep efficiency showed a significant main effect of Time only (Table 1).

### 3.2. EEG Microstate Parameters

Significant Group × Time interactions were observed for microstate C duration (F(1, 30) = 9.24, raw *p* = 0.005, η^2^p = 0.236; the effect remained significant after FDR correction), microstate C contribution (F(1, 30) = 4.68, raw *p* = 0.039, η^2^p = 0.135; the effect remained significant after FDR correction) and microstate A occurrence (F(1, 30) = 4.75, raw *p* = 0.037, η^2^p = 0.137; the effect remained significant after FDR correction).

Post hoc comparisons revealed selective alterations in EEG microstate dynamics following cTBS. For microstate C, mean duration increased significantly after active cTBS compared with the pre-intervention assessment (*p* < 0.05), and was significantly greater in the cTBS group than in the sham group at post-intervention (*p* < 0.05). The contribution of microstate C also increased significantly from pre- to post-intervention in the cTBS group (*p* < 0.05).

**Figure 1:**
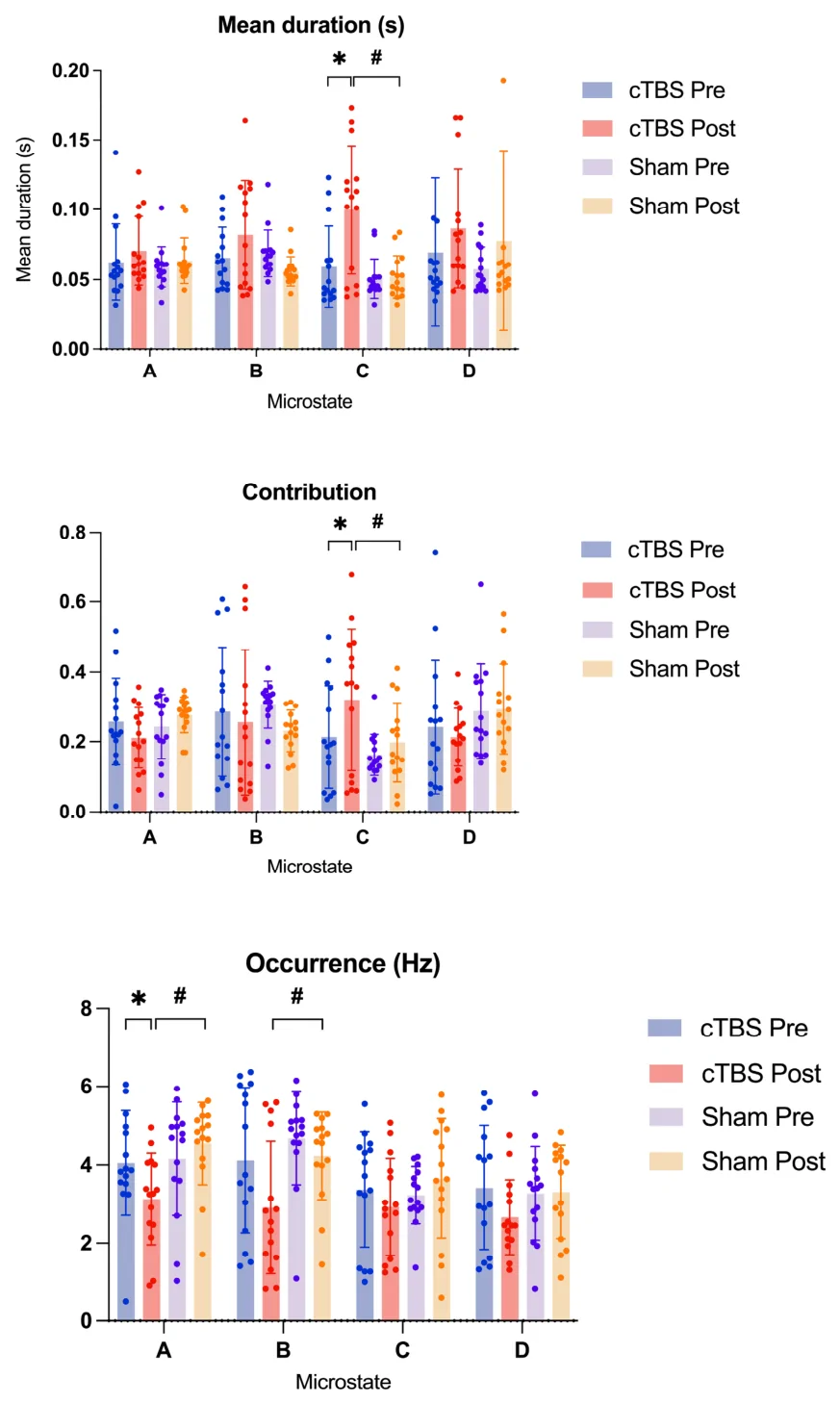
Selective modulation of EEG microstate duration, contribution, and occurrence by cTBS. Microstate parameters are shown for the active cTBS and sham groups at pre- and post-intervention. The upper, middle, and lower panels display mean duration, contribution, and occurrence, respectively, for microstates A–D. Active cTBS significantly increased microstate C duration and contribution and decreased microstate A occurrence. Microstate B occurrence was also significantly lower in the active cTBS group than in the sham group at post-intervention. Dots indicate individual values. * *p* < 0.05 for pre–post comparisons within the active cTBS group; # *p* < 0.05 for post-intervention comparisons between groups.

For microstate occurrence, microstate A showed a significant reduction following active cTBS (*p* < 0.05), with a lower post-intervention occurrence in the cTBS group than in the sham group (*p* < 0.05). Microstate B occurrence was also significantly lower in the cTBS group than in the sham group at post-intervention (*p* < 0.05). No significant differences were observed for the remaining microstate parameters (Figure 1).

### 3.3. EEG Microstate Transition Probabilities

**Figure 2:**
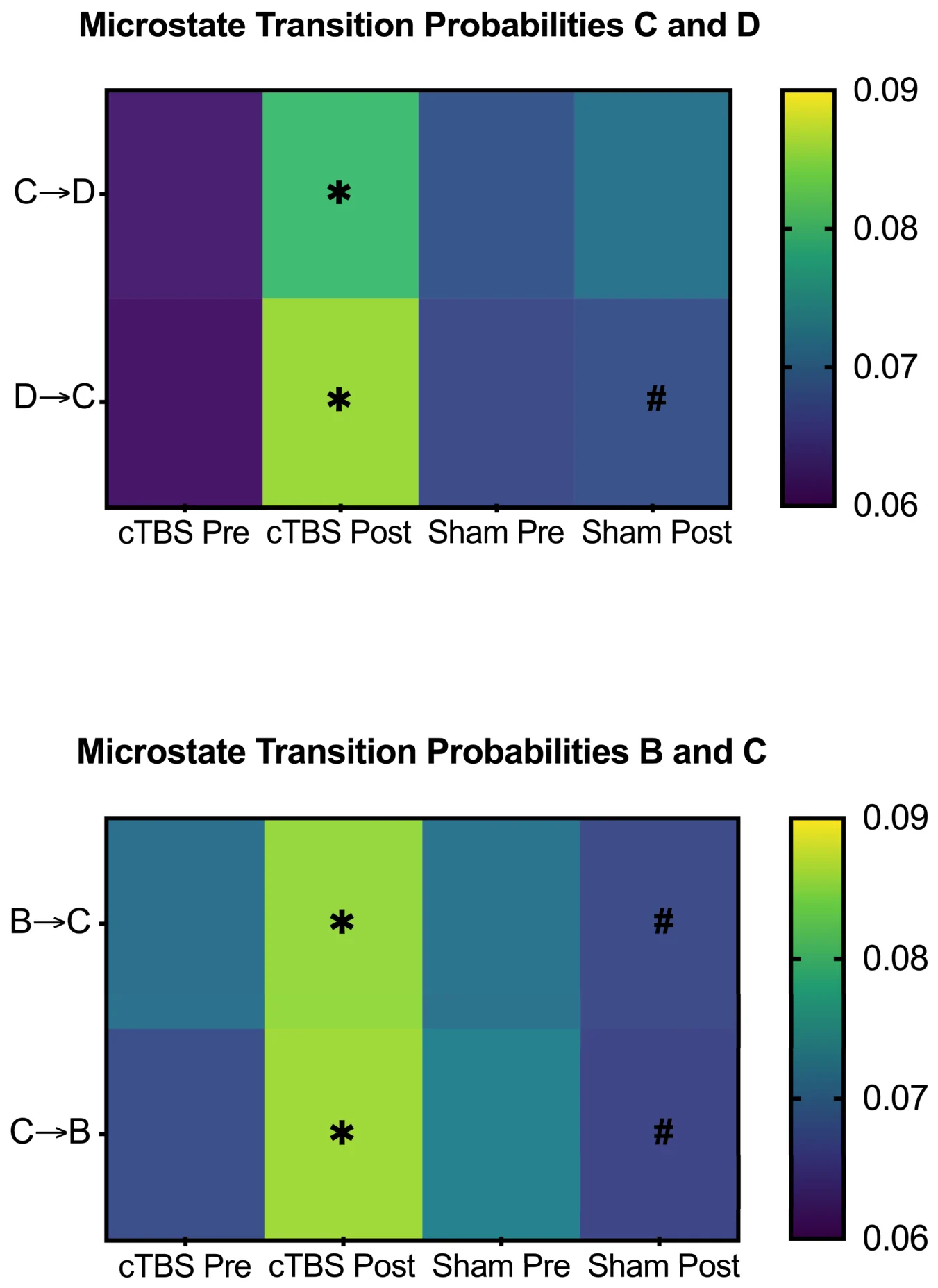
Changes in directional EEG microstate transition probabilities following cTBS intervention. Mean transition probabilities for selected directional transitions are displayed as heatmaps for the active cTBS and sham groups at pre- and post-intervention. The upper panel shows C to D and D to C transitions, whereas the lower panel shows B to C and C to B transitions. Warmer colors indicate higher transition probabilities. Active cTBS significantly increased the transition probabilities of C to D, D to C, B to C, and C to B. Post-intervention values for D to C, B to C, and C to B were significantly greater in the active cTBS group than in the sham group. * *p* < 0.05 for pre–post comparisons within the active cTBS group; # *p* < 0.05 for post-intervention comparisons between groups.

Significant Group × Time interactions were observed for directional transition probabilities from microstate D to C (F(1, 30) = 10.77, raw *p* = 0.003, η^2^p = 0.264; the effect remained significant after FDR correction), B to C (F(1, 30) = 8.12, raw *p* = 0.008, η^2^p = 0.213; the effect remained significant after FDR correction), and C to B (F(1, 30) = 8.39, raw *p* = 0.007, η^2^p = 0.219; the effect remained significant after FDR correction). Post hoc comparisons showed that active cTBS significantly increased the directional transition probabilities from microstate C to D, D to C, B to C, and C to B from pre- to post-intervention (all *p* < 0.05). At post-intervention, the transition probabilities for D to C, B to C, and C to B were significantly higher in the active cTBS group than in the sham group (all *p* < 0.05). No significant post-intervention between-group difference was observed for the C to D transition (Figure 2).

### 3.4. Sensitivity Analysis of EEG Microstate Transition Probabilities

Sensitivity analyses based on ΔTM values, which represent deviations of the observed transition probabilities from their expected values, yielded findings that were largely consistent with the primary OrgTM analyses. Significant Group × Time interactions for the D to C (F(1, 30) = 10.20, raw *p* = 0.003, η^2^p = 0.254; the effect remained significant after FDR correction), B to C (F(1, 30) = 7.73, raw *p* = 0.009, η^2^p = 0.205; the effect remained significant after FDR correction), and C to B (F(1, 30) = 8.45, raw *p* = 0.006, η^2^p = 0.220; the effect remained significant after FDR correction) transitions were observed in both the OrgTM and ΔTM analyses. In addition, a significant Group × Time interaction for the C to D transition emerged in the ΔTM analysis, although this interaction was not significant in the primary OrgTM analysis; nevertheless, the direction of change was consistent with the within-group increase observed in the raw transition probabilities. Specifically, the active cTBS group showed greater pre-to-post increases in these directional transitions than the sham group. No reversal of the estimated effects was observed after accounting for the expected transition structure. These findings indicate that the intervention-related alterations in microstate switching were not solely attributable to changes in the overall prevalence of the corresponding microstate classes and support the robustness of the primary transition results.

### 3.5. Effects of cTBS on Sports Performance

Mixed-design analyses revealed significant Group × Time interactions for lower-limb explosive power, 50 m sprint performance, and agility. Post hoc comparisons showed that the active cTBS group exhibited significant improvements in countermovement jump height (F(1, 30) = 8.00, raw *p* = 0.008, η^2^p = 0.195; the effect remained significant after FDR correction) and Illinois Agility Test (F(1, 30) = 6.57, raw *p* = 0.016, η^2^p = 0.180; the effect remained significant after FDR correction) performance following the intervention, whereas the sham group showed a significant pre-to-post change only in 50 m sprint time (adjusted *p* < 0.05).

Correlation analyses further indicated strong associations between sleep quality and sports performance. Lower PSQI scores, reflecting better sleep quality, were associated with greater countermovement jump height (r < −0.80, *p* < 0.05), whereas higher PSQI scores were associated with longer 50 m sprint and Illinois Agility Test completion times (r > 0.80, *p* < 0.05). These findings suggest that better subjective sleep quality was associated with superior explosive power, sprint performance, and agility (Table 2).

## 4. Discussion

Given the distinctive sleep-related challenges faced by athletes, the present study investigated whether a 7-day course of right dorsolateral prefrontal cTBS could improve subjective sleep quality and sport-related performance and whether these behavioral changes were accompanied by alterations in resting-state EEG microstate dynamics. Active cTBS improved PSQI global scores and selected aspects of sport-related performance. In parallel, EEG analyses showed selective changes in microstate C duration and contribution, microstate A occurrence, and several directional transition probabilities among microstates B, C, and D. Because canonical microstate classes have only approximate and context-dependent functional interpretations, these EEG findings should be interpreted primarily as changes in the temporal organization of scalp-level resting-state EEG activity, rather than as direct evidence of specific network-level mechanisms [10].

### 4.1. Efficacy of cTBS on Subjective Sleep Quality

Following the 7-day intervention, active cTBS produced significant reductions in the PSQI global score, with the largest improvements observed in sleep latency, subjective sleep quality, and habitual sleep efficiency. This pattern suggests that cTBS was associated with meaningful improvements in both sleep initiation and overall sleep quality in athletes with chronic insomnia. The present findings are broadly consistent with previous cTBS studies using polysomnography, which also demonstrated beneficial effects on objective sleep parameters in insomnia [10,11]. In athletic populations, however, the routine use of polysomnography may be less practical because multichannel monitoring, laboratory-based sleep recording, and first-night effects can interfere with habitual sleep behavior and reduce ecological validity [28]. By contrast, the PSQI offers a feasible, low-burden, and context-appropriate measure for capturing sleep-related changes in athletes. Therefore, the present results not only support the sleep-promoting effects of cTBS, but also highlight its practical relevance as a short-course neuromodulatory intervention for managing chronic insomnia in sports populations [29,30].

### 4.2. Selective Modulation of Resting-State Microstate Dynamics Following cTBS

Active cTBS induced selective rather than global alterations in resting-state EEG microstate dynamics, as reflected by increased duration and contribution of microstate C and reduced occurrence of microstate A. Recent evidence has demonstrated that a single session of theta-burst stimulation can modulate resting-state EEG microstates in healthy individuals [13]. The present findings extend these observations by demonstrating that a 7-day course of repeated therapeutic cTBS reshaped microstate organization in athletes with chronic insomnia. This pattern indicates that cTBS was associated with a selective modification of the temporal organization of canonical resting-state microstates, rather than a uniform redistribution across all microstate classes [31,32].

The most prominent effect involved microstate C. The concurrent increase in duration and contribution indicates that this microstate persisted for longer periods and occupied a greater proportion of the analyzed resting-state EEG after active cTBS [13]. Previous EEG-fMRI and microstate studies have associated microstate C with salience-related and interoceptive-autonomic processing; however, these functional correspondences are not exclusive and may vary according to the experimental context and microstate segmentation approach [31,32,33]. Therefore, the present findings should be interpreted primarily as evidence that cTBS altered the temporal expression of microstate C in athletes with chronic insomnia. This result is relevant to prior insomnia research reporting abnormalities in microstate temporal parameters, including altered microstate C dynamics, although direct comparisons should remain cautious because previous studies differed in sample characteristics and microstate classification procedures [34,35].

Microstate A occurrence was reduced following active cTBS, indicating that participants entered this microstate less frequently after the intervention. Microstate A has been associated with auditory and verbal/phonological processing in previous work [32,33]. Accordingly, the present result demonstrates a selective reduction in the temporal recurrence of a microstate class previously linked to sensory-verbal processing. However, the current data do not permit direct inferences regarding internally generated verbal thought, rumination, or other specific cognitive processes. Although microstate B occurrence was lower in the active cTBS group than in the sham group at post-intervention, the absence of a significant Group × Time interaction indicates that this finding should be interpreted as a post-intervention group difference rather than a robust intervention-specific effect.

Taken together, these findings indicate that cTBS was associated with selective changes in the persistence, temporal contribution, and recurrence of specific resting-state microstates. In conjunction with the observed improvement in subjective sleep quality, the results support the interpretation that cTBS modified the temporal organization of resting-state EEG activity in athletes with chronic insomnia [31,36].

### 4.3. Changes in Microstate Transition Probabilities Following cTBS

The transition-level findings extend the temporal microstate results and provide further evidence that active cTBS reshaped the sequential organization of resting-state brain activity in athletes with chronic insomnia. Whereas previous studies primarily characterized changes in the temporal properties of individual microstates [13], the present study further examined directional transition probabilities between brain states, providing additional insight into the dynamic syntax of spontaneous brain activity following repeated therapeutic cTBS. In addition to the increased duration and contribution of microstate C and the reduced occurrence of microstate A, active cTBS was associated with increased directional transitions among microstates B, C, and D, particularly C to D, D to C, B to C, and C to B. This pattern suggests that the intervention did not merely alter the temporal dominance of isolated microstates, but also modified the switching architecture through which large-scale brain states dynamically interact [37].

However, these transition results should be interpreted cautiously. Microstate transition probabilities can be sensitive to preprocessing choices, clustering stability, back-fitting procedures, and the relative prevalence of individual microstate classes. Moreover, the functional meaning of transitions between canonical microstates remains less established than that of the temporal parameters themselves, particularly in insomnia populations. Therefore, the present findings should not be taken as direct evidence of specific network interactions, such as salience-control or perceptual-internal processing coupling. Instead, they provide preliminary evidence that active cTBS may alter the temporal sequencing of resting-state EEG microstates in athletes with chronic insomnia [33,36].

Importantly, the sensitivity analyses based on ΔTM values strengthened this interpretation. Because ΔTM reflects deviations of the observed transition probabilities from their expected values, the preservation of significant Group × Time interactions for C to D, D to C, B to C, and C to B indicates that the primary OrgTM findings were not simply a secondary consequence of changes in the overall prevalence of the corresponding microstate classes [21]. In other words, the increased transition probabilities after active cTBS cannot be reduced to the fact that certain microstates occurred more or less often; rather, they suggest a genuine intervention-related reorganization of transition structure itself. The concordance between the OrgTM and ΔTM analyses therefore provides an important robustness check and supports the view that cTBS influenced both the stability of specific microstates and the dynamic rules governing their moment-to-moment switching [21,36]. One of the most notable findings of the present study was the enhanced bidirectional transitions between microstates C and D following active cTBS. Rather than reflecting prolonged engagement of a single functional state, increased C to D transitions may indicate more flexible temporal coordination between salience-related processing and executive control systems [23]. Such dynamic reorganization is consistent with emerging views that healthy brain function depends not only on the strength of large-scale networks but also on their capacity to rapidly switch between functional configurations.

Within the insomnia hyperarousal framework, the parallel improvement in sleep quality and alteration of microstate transitions may suggest that cTBS influences resting-state temporal organization in a direction consistent with reduced sleep-related arousal [38]. Together with the concurrent improvements in sleep quality, these findings suggest that the therapeutic effects of cTBS in athletes with chronic insomnia may involve a normalization of both the temporal persistence and transition architecture of resting-state brain states [35]. Rather than merely modifying the temporal characteristics of individual microstates, repeated therapeutic cTBS appeared to reorganize the transition architecture of spontaneous brain states. These findings suggest that adaptive brain-state switching, rather than isolated changes in microstate properties alone, may represent a more sensitive neurophysiological marker of treatment-related neural plasticity underlying sleep improvement in athletes with chronic insomnia.

However, because no mediation analysis was performed and no direct measures of cognitive or autonomic arousal were included, the present study cannot determine whether microstate transition changes contributed to sleep improvement. These EEG findings should therefore be regarded as exploratory electrophysiological correlates of cTBS response rather than mechanistic evidence. Future studies using larger samples, test–retest designs, source localization, functional connectivity analyses, or simultaneous EEG-fMRI (Functional Magnetic Resonance Imaging) will be needed to determine whether these transition-level changes correspond to specific large-scale network mechanisms.

### 4.4. Sleep-Associated Enhancement of Athletic Performance Following cTBS

In addition to improving sleep, active cTBS was associated with significant gains in athletic performance, including lower-limb explosive power and agility, with agility showing the largest improvement relative to baseline [39]. This pattern suggests that the benefits of cTBS in athletes with chronic insomnia may extend beyond sleep regulation and influence functional domains directly relevant to training and competition. Notably, correlation analyses demonstrated that better sleep quality was strongly associated with superior performance across all three measures, indicating a close link between sleep restoration and athletic capacity in this population [1,2,3]. These findings are consistent with previous evidence showing that adequate sleep supports motor recovery, sensorimotor integration, psychomotor speed, and the functional optimization of cortico-subcortical motor circuits [3,6,40].

When considered together with the observed changes in EEG microstate dynamics, the present findings further suggest that cTBS may improve performance through a broader reorganization of brain functional state regulation. Specifically, the improvement in sleep quality, the increased stability and contribution of microstate C, the reduced occurrence of microstate A, and the altered transition probabilities among microstates B, C, and D collectively indicate that cTBS may have promoted a more adaptive resting-state neural configuration. Such a shift may provide a more favorable neurophysiological basis for rapid information processing, motor coordination, and efficient behavioral output, which may be particularly relevant for tasks requiring agility and speed [41,42]. However, the present study was not designed to test whether EEG microstate alterations mediated improvements in sport-related performance. Therefore, the association among sleep improvement, microstate changes, and performance enhancement should be interpreted descriptively. It remains possible that improved sleep quality, nonspecific recovery effects, practice effects, or other unmeasured factors contributed to the observed performance gains. Future studies combining mediation models, larger samples, and task-based neurophysiological recordings during motor performance will be required to clarify whether resting-state EEG changes have predictive or mechanistic relevance for athletic performance outcomes.

### 4.5. Positioning cTBS Relative to iTBS and CBT-I

Compared with intermittent theta-burst stimulation (iTBS), which is classically associated with facilitatory-like effects, cTBS is generally associated with inhibitory-like after-effects, particularly when assessed over the motor cortex [43]. However, the magnitude and even the direction of TBS effects may vary across individuals, cortical targets, stimulation parameters, and baseline brain states [43,44]. Given that insomnia is frequently associated with hyperarousal and altered regulation of resting-state networks, cTBS may provide a theoretically relevant approach for modulating hyperarousal-related neural activity. Nevertheless, direct comparisons between cTBS and iTBS in insomnia populations remain limited, and future head-to-head trials are needed.

CBT-I (Cognitive Behavioral Therapy for Insomnia) remains the first-line behavioral treatment for chronic insomnia due to its robust clinical efficacy and long-term benefits. Rather than replacing CBT-I, cTBS may represent a complementary intervention by targeting neurophysiological mechanisms underlying hyperarousal and network dysregulation. Future studies combining cTBS with CBT-I may clarify whether neuromodulation can enhance or prolong therapeutic effects [45].

Rather than replacing CBT-I, cTBS may represent a potential adjunct delivered through a distinct neuromodulatory route. Because combined treatment was not evaluated in the present study, any additive or synergistic effects remain unknown. Future factorial or sequential trials should determine whether adding cTBS to CBT-I accelerates improvement, benefits individuals with an incomplete CBT-I response, or prolongs treatment effects.

### 4.6. Limitations

Several limitations should be considered when interpreting the present findings. First, sleep outcomes were assessed using the PSQI without polysomnography or actigraphy; therefore, the observed effects primarily reflect changes in subjective sleep quality, while potential alterations in objective sleep architecture remain to be examined [28,46]. Second, the relatively small sample consisted exclusively of young male university athletes, which may limit generalizability to other athletic populations [47]. Third, although a sham-controlled design and standardized assessments were used, blinding success was not formally evaluated, and training load, recovery status, and potential test-familiarization effects were not objectively quantified. In addition, outcomes were assessed only immediately after the 7-day intervention, leaving the durability of the effects uncertain [13]. Fourth, the right DLPFC was localized using the F4 position and stimulation intensity was determined relative to the motor threshold, both of which are commonly used approaches, although neuronavigation and individualized electric-field modeling may further improve targeting precision [48,49]. Finally, the EEG analyses were exploratory and involved multiple microstate measures. Although FDR correction and ΔTM sensitivity analyses were applied, replication in larger samples is warranted. Because mediation analysis and direct measures of arousal were not included, the relationships among EEG changes, sleep improvement, and sport-performance outcomes should be interpreted as associative rather than causal [13,50,51].

## 5. Conclusions

In conclusion, the present study demonstrates that a 7-day course of active cTBS applied over the right dorsolateral prefrontal cortex improved subjective sleep quality in athletes with chronic insomnia and was accompanied by improvements in sport-related performance. In parallel, active cTBS was associated with selective changes in resting-state EEG microstate temporal parameters and directional transition probabilities. Given the small sample size, the scalp-level nature of EEG microstate analysis, and the absence of mediation testing, these EEG findings should be interpreted as exploratory electrophysiological correlates of treatment response rather than evidence of a causal mechanism linking cTBS to sleep or performance improvements. Taken together, the findings support the feasibility of cTBS as a brief non-pharmacological intervention in athletes with chronic insomnia and provide a basis for larger mechanistic studies combining EEG microstates with source localization, connectivity analysis, or simultaneous EEG-fMRI.

## Figures and Tables

**Table 1:** PSQI scale and sub-component scores before and after cTBS intervention.

Index	Active cTBS Group		Sham Group	
	Pre	Post	Pre	Post
Subjective sleep quality	2.16 ± 0.65	1.15 ± 0.60 *^#^	2.24 ± 0.62	1.89 ± 0.58 ^#^
Sleep latency	2.77 ± 0.38	0.58 ± 0.32 *^#^	2.65 ± 0.42	1.42 ± 0.60 *^#^
Sleep duration	2.31 ± 0.52	1.63 ± 0.55 *^#^	2.38 ± 0.50	1.92 ± 0.53 ^#^
Habitual sleep efficiency	2.33 ± 0.48	0.72 ± 0.35 *	2.44 ± 0.47	1.36 ± 0.57 *
Sleep disturbances	2.54 ± 0.45	1.26 ± 0.62 *^#^	2.11 ± 0.64	1.69 ± 0.59 ^#^
Use of sleep medication	0.00 ± 0.00	0.00 ± 0.00	0.00 ± 0.00	0.00 ± 0.00
Daytime dysfunction	1.87 ± 0.63	1.40 ± 0.58	1.90 ± 0.61	1.51 ± 0.56
PSQI	13.98 ± 3.45	6.74 ± 2.75 *^#^	13.72 ± 3.40	9.79 ± 3.15 *^#^

* indicates a significant within-group difference between pre- and post-intervention (*p* < 0.05). ^#^ indicates a significant between-group difference at post-intervention (*p* < 0.05).

**Table 2:** Differences in sports performance before and after intervention.

Index	Active cTBS Group		Sham Group	
	Pre	Post	Pre	Post
CMJ (cm)	48.60 ± 3.20	52.10 ± 3.10 *^#^	47.90 ± 2.90	49.30 ± 3.40 ^#^
50 m sprint (s)	6.63 ± 0.28	6.32 ± 0.30 *	6.66 ± 0.28	6.33 ± 0.27 *
IAT (s)	16.01 ± 1.10	14.98 ± 0.90 *^#^	15.95 ± 1.30	15.60 ± 1.20 ^#^

* indicates a significant within-group difference between pre- and post-intervention (*p* < 0.05). ^#^ indicates a significant between-group difference at post-intervention (*p* < 0.05).

## Data Availability

Due to the privacy protection of sensitive information, we cannot disclose our data.

## Supplementary Materials

The following supporting information can be downloaded at: https://www.mdpi.com/article/10.3390/brainsci16080841/s1, Figure S1: CONSORT 2025 Flow Diagram; Table S1: CONSORT 2025 checklist of information to include when reporting a randomised trial.

## Abbreviations

The following abbreviations are used in this manuscript:

rTMS	Repetitive transcranial magnetic stimulation
cTBS	Continuous theta-burst stimulation
EEG	Electroencephalography
PSQI	Pittsburgh Sleep Quality Index
GFP	Global field power
CMJ	Countermovement jump
IAT	Illinois Agility Test
fMRI	Functional Magnetic Resonance Imaging
CBT-I	Cognitive Behavioral Therapy for Insomnia
