# Supplementary material for: Repeated Continuous Theta-Burst Stimulation Modulates Resting-State EEG Microstate Dynamics in Athletes with Chronic Insomnia
Source: Brain Sci. 2026 Aug 7;16(8):841. doi: 10.3390/brainsci16080841 (PMC13510399; doi:10.3390/brainsci16080841)
Supplement: Supplementary file 1 [file brainsci-16-00841-s001.zip › Figure S1.pdf]

Figure S1: CONSORT 2025 Flow Diagram

Flow diagram of the progress through the phases of a randomised trial of two groups (that is, enrolment, intervention allocation, follow-up, and data analysis)

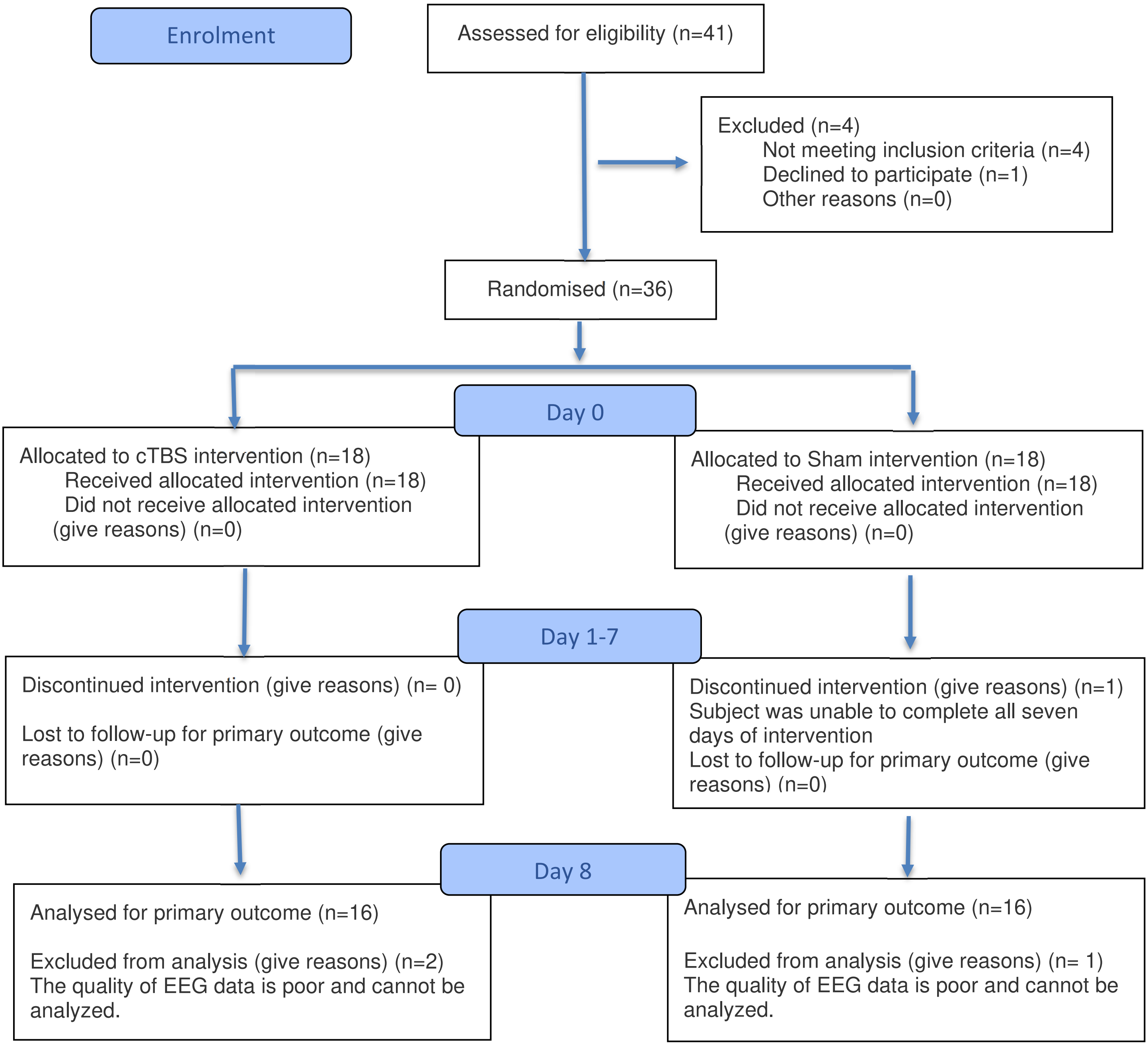

Citation: Hopewell S, Chan AW, Collins GS, Hróbjartsson A, Moher D, Schulz KF, et al. CONSORT 2025 Statement: updated guideline for reporting randomised trials. BMJ. 2025; 388:e081123. https://dx.doi.org/10.1136/bmj-2024-081123
© 2025 Hopewell et al. This is an Open Access article distributed under the terms of the Creative Commons Attribution License (https://creativecommons.org/licenses/by/4.0/), which permits unrestricted use, distribution, and reproduction in any medium, provided the original work is properly cited.
